# Lifestyle and Self-Perceived Quality of Life in Sports Students: A Case Study

**DOI:** 10.3390/ijerph19031598

**Published:** 2022-01-30

**Authors:** Juan Gavala-González, Amanda Torres-Perez, Ismael Gálvez-Fernández, José Carlos Fernández-García

**Affiliations:** 1 Department of Physical Education and Sports, University of Seville, 41013 Seville, Spain; jgavala@us.es; 2 Department of Didactics of Languages, Arts and Sport, University of Malaga, Andalucía-Tech, IBIMA, 29071 Malaga, Spain; jcfg@uma.es

**Keywords:** physical activity, lifestyle, quality of life, adolescents

## Abstract

Adolescence has been considered a crucial stage for the adoption of healthy habits such as physical activity. In addition, numerous research studies have shown that physical activity is a positive factor for health behaviors and quality of life. In this sense, the aim of this study was to examine the relationship between physical activity levels and perceived quality of life in a sample of students studying physical activity and sport. This is a descriptive observational study with a population of physical activity and sports students from the San Pablo High School in Seville, Spain. The participants (*N* = 86), with a mean age of 18.56 ± 1.88 years, were pursuing professional qualifications in Physical Activity and Sport. They were administered the short version of the International Physical Activity Questionnaire (IPAQ-SF) and the SF-36 questionnaire on perceived health status. For data analysis, a correlation analysis (Spearman’s rho) was performed. The results indicate that students attending sports-related vocational training programs engaged in more physical activity (96.9%) than the population average suggested by the World Health Organization (20%). In addition, a direct relationship was shown between participating in physical activity and perceived health. Depending on the intensity of the physical activity, students found less physical interference (IPAQ Vigorous—Physical Function (rho = 0.252; *p* = 0.019); IPAQ Sedentary—Bodily Pain (rho = 0. 223; *p* < 0.039); IPAQ Total—Physical Function (rho = 0.256; *p* = 0.018)) and emotional interference (IPAQ Moderate—Emotional Role (rho = 0.237; *p* = 0.028)) when performing exercise or activities of daily life; therefore, physical activity appears to be beneficial for self-perception of quality of life.

## 1. Introduction

Defining the life phase of adolescence is not an easy task for science because it encompasses elements of biological growth and important social role transitions, which have been changing throughout history and particularly at the end of the twentieth century. The earlier biological initiation of puberty has accelerated the onset of adolescence in almost all populations, while an understanding of continued growth has raised its final age well into the twenties, although the World Health Organization (WHO) continues to define adolescence as the period of growth that occurs after childhood and before adulthood, between the ages of 10 and 19 years [1,2].

Adolescence is a stage of human evolutionary development in which there is a continuous process of highly relevant changes in physical, psychological and social maturation [3]. These changes have a considerable influence on the development and consolidation of aspects such as individual identity, the configuration of self-concept and its dimensions, daily habits and lifestyle [4,5,6].

One of the most important aspects during this stage, which is essential for the appropriate overall development of the adolescent, is the adoption of healthy lifestyle habits, understood as health patterns, behaviors, beliefs, knowledge and actions carried out to maintain, restore or improve health [7,8]. This is because it is during adolescence when habits and behaviors that will be maintained throughout life are developed and established [9,10,11,12].

There is currently widespread concern about the increase in sedentary behaviors among the population, especially among young people [13,14,15,16]. Free time is spent on activities such as watching television, surfing the Internet or using cell phones, while physical activity is neglected [6,13,14]. This has strong negative repercussions for health status, and may considerably increase problems arising from inactivity, such as being overweight or obese, thus seriously affecting quality of life [15,16,17,18,19,20].

However, despite the various proven benefits of physical activity [21,22,23,24,25], only 30–40% of young people are sufficiently active, according to public health recommendations [26]. These guidelines recommend performing at least 60 min a day of moderate or vigorous activity and working on muscular strength and flexibility for a minimum of two days a week, as this seems to be adequate to prevent problems associated with inactivity [15,27,28,29]. According to the WHO, around 80% of the world’s adolescents have an insufficient level of physical activity [29,30].

Numerous studies support a clear, positive relationship between physical activity and general health [21,25,31,32,33,34,35], as well as physical [21,36], social [34,37] and psychological health [38], which is why physical activity should be a fundamental activity in the leisure time of adolescents [39,40,41]. Several studies [17,18,25,36,39] have shown the multiple benefits of regular and adequate physical activity on health behaviors and quality of life, as manifested by improved mood, greater resistance to stress, better physical condition and a better self-perception of health and quality of life [5,42,43,44,45,46]. In addition, adolescents who engage in more active behaviors also perceive themselves as having better health and greater life satisfaction [46,47,48,49], while those who engage in more sedentary behaviors, such as watching television or spending time on their cell phones, are associated with problems such as poorer mental health [28,50]. This relationship between physical activity and self-perceived health is consistent throughout life. Thus, lifestyle during adulthood may be affected by physical activity behaviors adopted during the early stages of life [50]. 

Finally, studies using the International Physical Activity Questionnaire (IPAQ) to evaluate physical activity and “The SF-36 health status questionnaire” for quality of life show significant relationships between these variables, which implies that greater physical activity is associated with better self-perceived health [45,51,52,53]. 

The main purpose of the present study was to determine the relationship between different levels of physical activity and self-perception of quality of life in a sample of adolescent students attending vocational education programs in physical activity and sports, because the practice of physical activity is considered a promoter of good health and quality of life [33,34,54].

## 2. Materials and Methods

### 2.1. Design and Participants

This was an observational, descriptive study using a cross-sectional correlational methodology, in which questionnaires served as a tool for data collection [55]. All measurements were performed on a single occasion. The study sample comprised the entire population of intermediate or advanced vocational training students enrolled in a Physical Activity and Sport program at the IES San Pablo High School in Seville, Spain, for a total of 86 participants—63 men and 23 women—with a mean age of 18.56 ± 1.88 years (Table 1).

After the initial selection, the participants were informed of the nature of the study, indicating that their anonymity would be maintained at all times, following the ethical standards for Sport and Exercise Science Research [56] and the principles set out in the Declaration of Helsinki [57], which define the ethical guidelines for research involving human subjects. The University of Malaga assigned the identification number 65-2020-H, which is registered with the Ethics Committee. The participants provided written informed consent, and throughout the intervention and afterward, we acted under the provisions of Organic Law 3/2018, of 5 December, on the Protection of Personal Data and Guarantee of Digital Rights, regarding the protection of personal data under Spanish law. After signing the informed consent form, the physical activity (IPAQ-SF) and the health-related quality of life (SF-36) questionnaires were administered.

### 2.2. Tools

To assess physical activity levels, we used the International Physical Activity Questionnaire—short version (IPAQ-SF), which consists of seven questions with acceptable measurement properties for monitoring physical activity levels in different environments and also reports the number of METs [58]. 

This questionnaire comprises seven questions about the frequency, duration and intensity of activity (moderate or vigorous) performed in the last seven-day period [59], and allows different levels of physical activity to be established:

- High: accumulating a total of at least 3000 MET/week.
- Moderate: exceeding 600 MET/week.
- Low: less than 600 MET/week.

The SF-36 health status questionnaire was used to measure self-perceived quality of life. This questionnaire consists of 36 items that report both positive and negative states of health and form 8 dimensions: physical function, social function, physical role, emotional role, mental health, vitality, bodily pain and general health [60].

For the evaluation of this questionnaire, the response options for each item form Likert-type scales assessing intensity or frequency, ranging from 3 to 6 depending on the item. The score for each item is coded and transformed into a scale ranging from 0 (worst state for that dimension) to 100 (best state), using the scoring manual of the Spanish version of the SF-36 health questionnaire [60]. In this way, each of the dimensions is scored between 0 and 100 points.

Both the IPAQ and the SF-36 are two approved questionnaires whose reliability and validity have been tested in different studies [61,62,63].

### 2.3. Data Analysis

All statistical analyses were performed with IBM SPSS Statistics 25 (IBM Corp., Armonk, NY, USA). The level of significance was set at *p* < 0.05. The fit of the different variables to the normal distribution was assessed by both graphic procedures (QQ plot) and the Kolmogorov–Smirnov test. The results showed that the study variables did not follow normality.

To verify the existence of significant relationships between the physical activity and health perception variables, correlation analysis (Spearman’s rho) was performed to measure the degree of association between the related quantitative variables.

## 3. Results

The descriptive analysis of the study variables (Table 2), the levels of physical activity (IPAQ-SF) and the different dimensions of self-rated quality of life (SF-36) are shown in the following table. 

With respect to self-perceived general health, 64.0% of the participants reported very good or excellent health, 33.7% said they were in good health and only 2.3% of the participants said they were in fair health. None of them said they were in poor health. 

For physical activity levels according to the IPAQ-SF questionnaire, 90.7% of the participants had a high level of physical activity, indicating that they accumulated at least 3000 METweek, and 9.3% had a moderate level, exceeding 600 MET/week. None of the participants, therefore, had a low level of physical activity.

When we consider differences according to sex and physical activity levels, we can see that, in general, both men and women considered to be vigorous in their activity reach significantly higher levels of total physical activity than those considered to be moderate. (Table 3). Practically all of the variables that show statistically significant differences present the size of the effects, calculated from the Hedges’ G for a paired sample, as being greater than 0.8. Therefore, the differences are statistically significant and clinically relevant.

The correlations between the different physical activity variables and the perceived quality of life dimensions are presented in Table 4.

**Figure 1:**
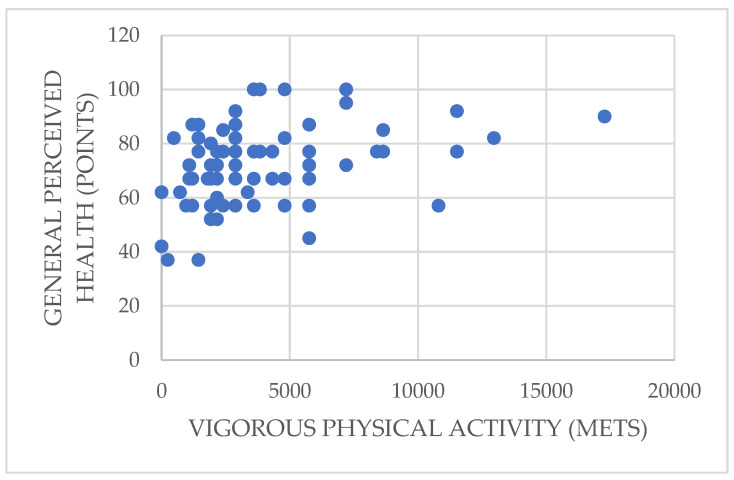
Relationship between vigorous physical activity and general perceived health.

Focusing on the most significant correlations found in the study, Figure 1 shows how vigorous physical activity has a highly significant, direct relationship with the participants’ general perceived health (rho = 0.343; *p* = 0.001 < 0.01), which implies that those who perform more vigorous activity perceive themselves to have a better health status. 

**Figure 2:**
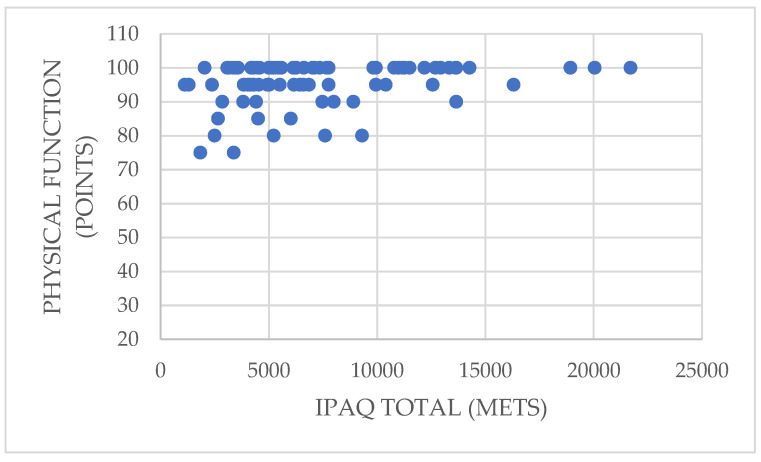
Relationship between total physical activity and the physical function dimension.

In addition, vigorous physical activity shows a significant direct correlation with the physical function dimension (rho = 0.252; *p* = 0.019 < 0.05). Similarly, the total physical activity variable is also directly related to physical function (rho = 0.256; *p* = 0.018 < 0.05), which implies that participants who performed more vigorous physical activity and those who accumulated a greater amount of total physical activity had better physical function, meaning that they had fewer health limitations when performing moderate and vigorous activities (Figure 2).

**Figure 3:**
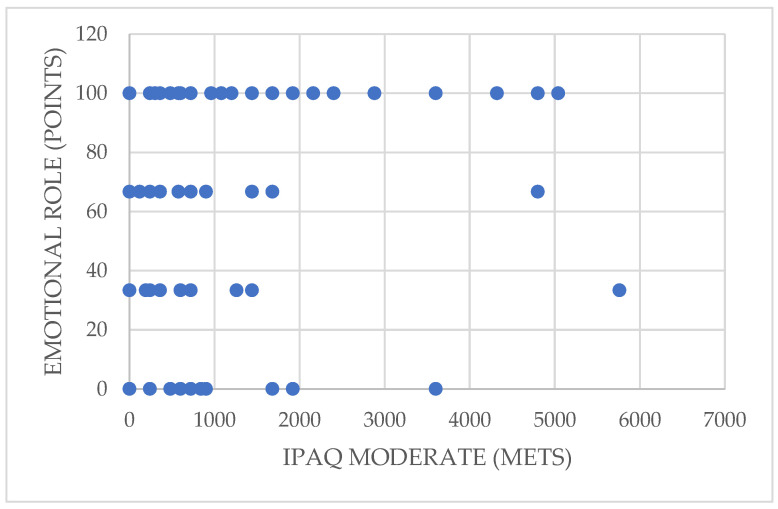
Relationship between moderate physical activity and the emotional role dimension.

Figure 3 illustrates the correlation between participation in moderate physical activity and the dimension of perceived quality of life called emotional role (rho = 0.237; *p* = 0.028 < 0.05). This shows that higher levels of moderate-intensity physical activity are associated with a better emotional role, i.e., that emotional problems in those who engage in a greater amount of moderate physical activity interfere less in their daily activities.

**Figure 4:**
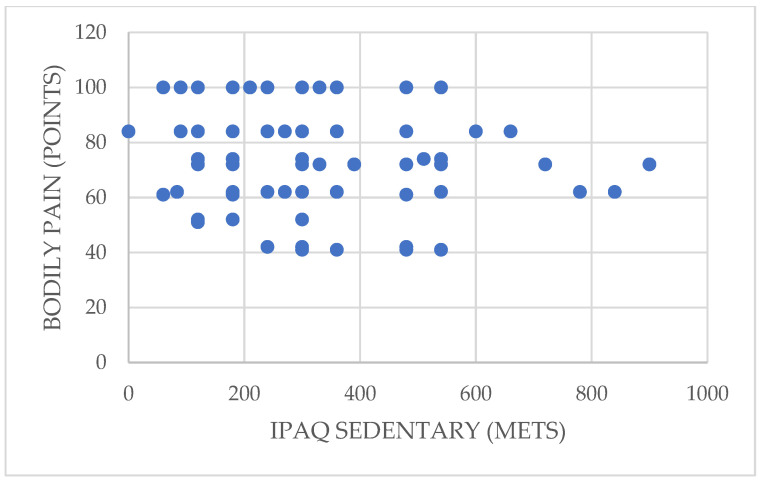
Relationship between sedentary activity and bodily pain.

On the other hand, sedentary activity shows a significant inverse correlation with bodily pain (rho = −0.223; *p* = 0.039 < 0.05). Thus, greater time spent on sedentary activities resulted in greater bodily pain in the participants (Figure 4).

**Figure 5:**
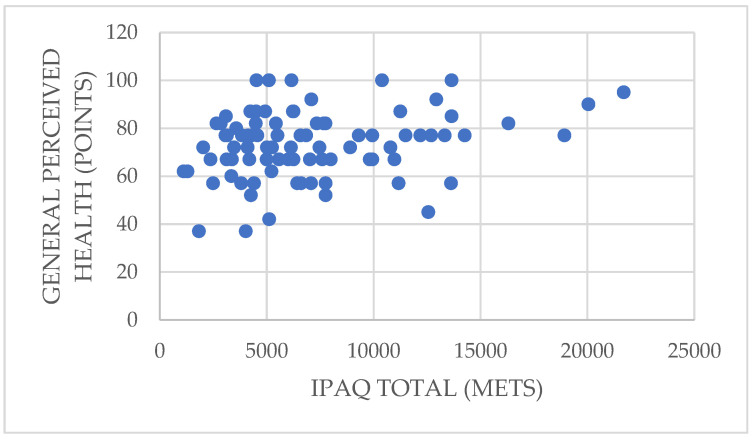
Relationship between total physical activity and general perceived health.

Finally, Figure 5 presents the relationship between the total physical activity variable and general perceived health. There is a very low but positive, non-significant relationship (rho = 0.209; *p* = 0.053 < 0.05), indicating that those who performed a greater amount of weekly physical activity perceived themselves to have a better general health status.

## 4. Discussion

The present study was undertaken to determine the possible effects that participation in regular physical activity can have on perceived health in a sample of adolescents; the new findings are related to the characteristics of the population, comprised of adolescents and young adults enrolled in studies related to physical activity and sport and who at a given time will be future promoters of physical activity. The variables observed were associated with healthy lifestyle habits and behaviors, since adolescence is the stage in which behaviors are established that will have repercussions on the health status of individuals throughout their lives [9,10,11,12]. 

Numerous studies have shown that physical activity provides multiple benefits for adolescents [21,25,31,32], ranging from improved mood and stress levels to better self-perceived health and quality of life [5,28,35,48,49,64,65].

An analysis of various studies assessing the perception of quality of life or performance of physical activity in adolescents shows that 90.9% of adolescents perceive themselves to be in good health [66]. The results of our study, however, are even better, with 97.8% of the students perceiving themselves to be in good to excellent health, and with regard to physical activity, all the participants in the study reported a high or moderate level. Concerning physical activity, 40.8% of adolescents in Spain do not engage in physical activity [67], and only 30–40% of young people are sufficiently active, bearing in mind the recommendations [26]. According to the WHO, around 80% of adolescents have an insufficient level of physical activity [30]. 

To analyze the different variables according to sex, we can take as a reference various studies that have shown that men are more active than women [67] and that women show worse results in terms of subjective health compared to men of the same age [68]. The results of our study are consistent with those of these previous studies, as the men were more active and perceived themselves to be in better health than the women.

Several studies have also shown that subjects who report greater physical activity perceive themselves to have a better health status, while less-active subjects report greater bodily pain [5,35,48,64,69]. Along the same lines, the results obtained in the present study are similar since those adolescents who performed more physical activity throughout the week had a better health status and better physical function, which implies that they had fewer health limitations when performing different physical activities. Those who were more inactive reported greater bodily pain.

Taking into account intensity as a differentiating parameter of physical activity, interesting relationships were found between engaging in physical activity and the different dimensions of quality of life. Thus, participants who performed more vigorous physical activity showed a better general health status and better physical function, while those with a moderate level of physical activity showed less interference from emotional problems in daily activities, which is in agreement with other studies [5,35,48,64].

A limitation of the study is that the results obtained in the correlation analyses show a weak association, since rho is <0.3, so we must take them with caution, even though they are statistically significant correlations.

## 5. Conclusions

In conclusion, the new findings of the present study are related to the characteristics of the population studied, being a school population of young adults enrolled in studies related to physical activity and sport and who, at a given time, will be future promoters of physical activity. Furthermore, in the future, this could allow us to analyze the existence of a relationship between different levels of physical activity students and the practice of physical activity. On the other hand, we must bear in mind that since this is a case study where normality is not satisfied, there is a certain limit to the generalization of the results obtained.

In this sense, students pursuing intermediate or advanced vocational training qualifications in physical activity and sport were very active, since they had extremely high mean METs, and more than 95% of the subjects evaluated associated their good physical condition with a better perception of their health status and quality of life.

This study has therefore shown physical activity to be beneficial for self-perceived quality of life. Those students who were more physically active reported a better self-perceived health status. In addition, the intensity of the physical activity was related to various perceived quality of life dimensions, including better physical function, emotional role and bodily pain, indicating that students who engaged in more intense physical activity experienced fewer health limitations or emotional problems when performing exercise or carrying out daily activities, while the more sedentary subjects reported greater bodily pain that interfered with their daily activities. 

Based on our results, we recommend that adolescents follow the WHO recommendations and engage in physical activity to improve their physical condition, lifestyle and self-perceived quality of life. 

## Figures and Tables

**Table 1:** Characteristics of the sample.

Age (Years)		18.56 ± 1.88
Sex (%)	Men	73.3
	Women	26.7
Degree (%)	Intermediate vocational training	41.9
	1st year of advanced vocational training	30.2
	2nd year of advanced vocational training	27.9

**Table 2:** Descriptive analysis of the study variables.

Variables	Results		
	General (*n* = 86)	Women (*n* = 23)	Men (*n* = 63)
IPAQ Walking (METs)	2013.8 ± 1951.4	2234.0 ± 2193.8	1933.5 ± 1867.7
IPAQ Moderate (METs)	1300.7 ± 1284.7	1093.0 ± 1325.4	1376.6 ± 1271.8
IPAQ Vigorous (METs)	3833.0 ± 3060.96	3240.0 ± 2309.1	4049.5 ± 3282.4
IPAQ Total (METs)	7147.6 ± 4282.7	6567.0 ± 3346.4	7359.6 ± 4582.7
IPAQ Sedentary (METs)	304.8 ± 181.2	279.1 ± 160.9	314.2 ± 188.5
Physical Function (Pts)	95.9 ± 6.2	95.43 ± 6.7	96.0 ± 6.1
Physical Role (Pts)	76.7 ± 29.2	73.9 ± 30.6	77.8 ± 28.8
Bodily Pain (Pts)	75.2 ± 18.4	73.1 ± 21.4	76.0 ± 17.2
General Perceived Health (Pts)	73.1 ± 13.8	70.0 ± 11.1	74.3 ± 14.6
Vitality (Pts)	65.1 ± 15.1	61.1 ± 18.4	66.6 ± 13.6
Social Functioning (Pts)	83.9 ± 21.0	73.9 ± 23.2	87.5 ± 19.1
Emotional Role (Pts)	75.6 ± 35.9	50.7 ± 41.3	84.7 ± 29.2
Mental Health (Pts)	70.8 ± 17.0	61.9 ± 17.0	74.1 ± 15.9

METs = Metabolic Equivalents of Task; Pts = points.

**Table 3:** Comparison of means of physical activity according to sex and physical activity levels (moderate or vigorous).

	Physical Activity Levels	Total Mets	*p*-Value	Effect Size
GENERAL (*n* = 86)	MODERATE (*n* = 8)	2082.0 ± 630.98	*p* = 0.00	1.40
	VIGOROUS (*n* = 78)	7667.2 ± 4155.9		
WOMEN (*n* = 23)	MODERATE (*n* = 2)	2433.0 ± 84.9	*p* = 0.00	1.43
	VIGOROUS (*n* = 21)	6960.7 ± 3232.1		
MEN (*n* = 63)	MODERATE (*n* = 6)	1965.0 ± 700.2	*p* = 0.00	1.39
	VIGOROUS (*n* = 57)	7927.4 ± 4445.4		

**Table 4:** Correlations between the physical activity variables and perception of quality of life (Spearman’s rho).

		PF	PR	BP	GPH	V	SF	ER	**MH**
IPAQ Moderate (METs)	Correlation coefficient	0.085	−0.019	0.154	−0.023	0.027	0.016	0.237 *	0.131
	Sig. (bilateral)	0.438	0.863	0.157	0.831	0.807	0.886	0.028	0.228
IPAQ Vigorous (METs)	Correlation coefficient	0.252 *	−0.006	0.092	0.343 **	0.031	−0.022	0.055	0.037
	Sig. (bilateral)	0.019	0.953	0.401	0.001	0.778	0.837	0.617	0.735
IPAQ Total (METs)	Correlation coefficient	0.256 *	−0.011	0.107	0.209	0.015	−0.040	0.110	−0.017
	Sig. (bilateral)	0,018	0.921	0.328	0.053	0.894	0.714	0.314	0.874
IPAQ Sedentary (METs)	Correlation coefficient	−0.045	0.009	−0.223 *	−0.100	−0.096	0.006	−0.051	0.008
	Sig. (bilateral)	0.682	0.932	0.039	0.359	0.379	0.958	0.638	0.942

METs = Metabolic Equivalents of Task; PF = Physical Function; PR = Physical Role; BP = Bodily Pain; GPH = General Perceived Health; V = Vitality; SF = Social Function; ER = Emotional Role; MH = Mental Health; * = *p* < 0.05; ** = *p* < 0.001.
